# Insulin and cancer: a tangled web

**DOI:** 10.1042/BCJ20210134

**Published:** 2022-03-04

**Authors:** Brooks P. Leitner, Stephan Siebel, Ngozi D. Akingbesote, Xinyi Zhang, Rachel J. Perry

**Affiliations:** 1 Departments of Cellular and Molecular Physiology, Yale School of Medicine, New Haven, CT, U.S.A.; 2 Departments of Internal Medicine, Yale School of Medicine, New Haven, CT, U.S.A.; 3 Departments of Pediatrics, Yale School of Medicine, New Haven, CT, U.S.A.

**Keywords:** cancer metabolism, diabetes, immunometabolism

## Abstract

For a century, since the pioneering work of Otto Warburg, the interwoven relationship between metabolism and cancer has been appreciated. More recently, with obesity rates rising in the U.S. and worldwide, epidemiologic evidence has supported a link between obesity and cancer. A substantial body of work seeks to mechanistically unpack the association between obesity, altered metabolism, and cancer. Without question, these relationships are multifactorial and cannot be distilled to a single obesity- and metabolism-altering hormone, substrate, or factor. However, it is important to understand the hormone-specific associations between metabolism and cancer. Here, we review the links between obesity, metabolic dysregulation, insulin, and cancer, with an emphasis on current investigational metabolic adjuncts to standard-of-care cancer treatment.

## Introduction: obesity and cancer epidemiology

Currently, the Centers for Disease Control have identified thirteen tumor types of which overweight and obesity increase risk [1–27] (Table 1). In most of these, excess weight also worsens prognosis. It should be noted that there may be a sampling bias in the link between obesity and cancer, though it is complex: a meta-analysis by Fagan et al. [28] demonstrated that obesity was associated with a decreased likelihood of screening for cervical and colorectal cancer, an increased likelihood of screening for prostate cancer, and no difference in rates of screening for breast cancer. Interestingly, there are other tumor types in which overweight and/or obesity may confer an improved response to treatment: in both lung cancer [29–30] and melanoma [31–33], as well as in pooled analyses of patients with any tumor treated with immune checkpoint inhibitors [34,35], outcomes were improved in overweight/obese subjects. There appear to be opposing continua of both tumor immunogenicity and association with obesity: in general, more immunogenic tumors appear to be less positively associated with overweight and obesity, and vice versa. In this review, we concentrate on tumors positively correlated with obesity, and the role of insulin in driving tumor progression, while recognizing that no monolith regarding the relationship between metabolism and cancer exists.

**Table 1.** Cancers associated with obesity in humans (adapted from [1])

Postmenopausal breast
Colorectal
Endometrial/uterine
Esophageal adenocarcinoma
Gallbladder
Gastric
Hepatocellular
Meningioma
Multiple myeloma
Ovarian
Pancreatic
Renal
Thyroid

## Obesity's link to insulin resistance and hyperinsulinemia

### Obesity linked to high insulin

A critical consequence of excess adiposity is insulin resistance, which has been thoroughly reviewed elsewhere [36–38]. There exists a wide spectrum of insulin resistance, where an insulin sensitive individual will have low basal and postprandial insulin concentrations, an insulin resistant individual will have hyperinsulinemia in both settings, and an individual with overt type 2 diabetes, whose pancreatic beta cells cannot properly secrete insulin in response to elevated glucose, presents with hyperglycemia without hyperinsulinemia [39]. A mildly insulin resistant individual will have obesity with or without hyperglycemia, but elevated insulin concentrations in the basal and postprandial state. Therefore, insulin resistant individuals have a decreased capacity to store plasma glucose as muscle and liver glycogen and suppress hepatic gluconeogenesis in response to insulin, commonly resulting in simultaneous hyperinsulinemia and hyperglycemia [38].

The molecular mechanism of insulin resistance, and thus elevated insulin levels, is downstream of the insulin receptor (IR) in at least muscle, liver, and adipose tissue, and may be the result of the accumulation of ectopic lipids, including ceramides and/or diacylglycerols, in these tissues. Several alternative mechanisms for the induction of insulin resistance in obesity are also supported by the literature. Elevated non-esterified fatty acids, branch-chained amino acids, and glucose have all been reported in the setting of obesity, each of which can contribute to nutrient-induced insulin resistance through various purported mechanisms [36]. Systemic and tissue-specific inflammation has also been implicated as a mechanistic link between obesity and insulin resistance, with interleukin-6 and c-Jun N-terminal kinase (JNK) signaling as key mediators [40–42]. Thus, it has become apparent that both the local and systemic environment can play substantial roles in the context of insulin resistance.

### Nuanced definition of obesity (body weight versus adiposity)

Though obesity is often defined epidemiologically by excess weight (typically BMI > 30 kg/m^2^), there are shortfalls to this approach in metabolic disease and cancer. BMI appears to relatively underestimate body fat percentage in certain populations [43,44], and overestimate in others [45]. Nonetheless, at large scale, risk of morbidity and mortality has been well described with BMI representing a proxy for excess adiposity. However, other surrogates for adiposity, such as waist circumference, waist-hip ratio [46], skin-fold measurements, medical imaging such as dual-energy x-ray absorptiometry (DXA) [47], computed tomography (CT) [48,49], or magnetic resonance imaging (MRI) provide much more accurate measures of body fat. In addition, medical imaging allows for the distinction between visceral and subcutaneous adiposity. Though visceral fat is only ∼5% and 3% of total adipose tissue for men and women, respectively [50], it confers a greater deleterious consequences for metabolic disease and cancer than excess weight alone [51]. In addition, visceral fat content is one of the strongest independent predictors of insulin resistance and hyperinsulinemia [52].

Few mechanisms have been explored in humans that interrogate how visceral adiposity modulates tumor biology [51,53,54]. As alluded to earlier in this review, obesity appears to be protective for survival in lung cancer. Recent work from our group tested the hypothesis that obesity as defined by BMI would uncover different immunometabolic characteristics of tumors than using visceral adiposity as a readout of metabolic health [48]. We demonstrated that when tumor gene expression analyses were performed on high versus low BMI patients, there were more differentially expressed genes with beneficial prognosis, including *CBX6*, *TOX3*, and *TMPRSS2* in patients with high BMI, consistent with BMI having a protective effect. However, high visceral adiposity versus low visceral adiposity analyses demonstrated an opposite effect on prognosis: expression of detrimentally prognostic genes (as determined from the PRECOG database [55]) including *KRT6A*, *FEM1B*, and *S100A2*, reveal that visceral adiposity, the more deleterious component of excess body mass, is associated with vastly different transcriptional profiles within the tumors. The mechanistic links of visceral adiposity to these transcriptomic profiles remain to be uncovered. In addition to altered transcriptomics between BMI and visceral adiposity comparisons, increased glucose uptake within lung tumors was positively correlated with visceral fat content, but not BMI, providing support for more nuanced relationships between body composition, metabolism, and prognosis than simply relying upon BMI. Other research has implicated adipose-derived inflammatory mediators (including IL-6, and IL-1β, but not MCP1) [53], altered amino acid metabolism (including serine/glycine and tryptophan metabolism) [56], or reactive oxygen species [57] as potential mediators of the link between body composition and cancer progression. These findings among others have led some to interrogate the concept of metabolically healthy obesity, and metabolically unhealthy leanness in metabolic disease and cancer [58–61].

### Nuanced definition of obesity (metabolically healthy obesity)

Though there is no clear definition of metabolically healthy obesity, the concept is that a person with a BMI greater than 30 kg/m^2^ can have normal blood glucose, triglycerides, cholesterol, and blood pressure, and that these individuals, though obese, may not have elevated risk for disease [62]. However, large epidemiological studies have shown that metabolically healthy obese individuals have greater all-cause mortality than metabolically healthy lean individuals [63], and that metabolically healthy obese individuals have greater odds of developing cancer than metabolically healthy lean individuals [64]. Conversely, there exist individuals with a BMI between 18.5 and 25 kg/m^2^ who nevertheless exhibit elevated cardiometabolic risk factors with decreased skeletal muscle mass and elevated visceral fat mass, referred to as metabolically unhealthy normal-weight individuals [65], who have higher risk for diabetes [66], a three-folder high risk of all-cause mortality/cardiovascular disease [67], and an increased risk of cancer [58].

### Nuanced definition of obesity (sarcopenic obesity and cachexia)

**Figure 1.**
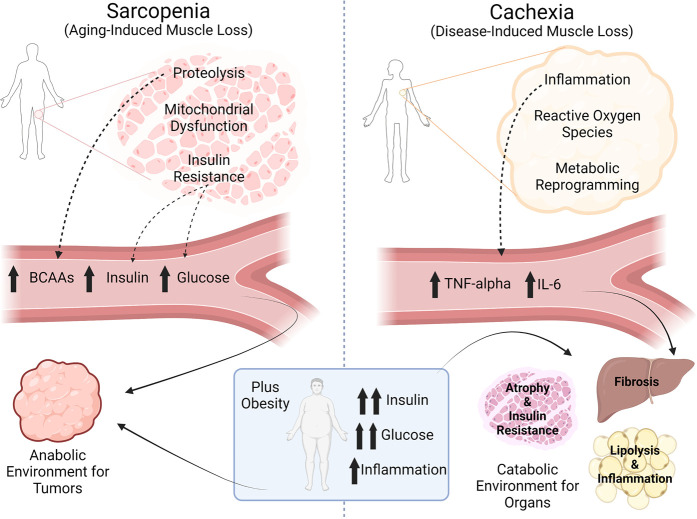
Sarcopenia creates an anabolic environment for tumors, while cancer cachexia creates a catabolic environment for organs. Made in BioRender.com.

Sarcopenic (loss of muscle mass, often associated with ageing) obesity is the concept that an individual can be meet clinical definitions of obesity (BMI > 30 kg/m^2^) and simultaneously exhibit skeletal muscle wasting [68,69]. Sarcopenic skeletal muscles are known to be insulin resistant even in the setting of low whole-body fat stores [70], and considering that skeletal muscle is a primary site of both insulin action and glucose uptake/storage [71–75], sarcopenia contributes significantly to systemic metabolic syndrome [76]. Numerous studies have shown detrimental epidemiological consequences of sarcopenic obesity on cancer incidence, progression, and survival, with the largest influence on cancer incidence [53,54,69,77–81]. Likely mechanisms of sarcopenia-associated insulin resistance include reduced mitochondrial function (i.e. the ability to oxidize metabolites) [82,83], reduced skeletal muscle mass and thus reduced skeletal muscle glucose disposal [53,84], as well as protein wasting that involves the release of deleterious metabolites [85] (Figure 1). For example, elevated branched-chain amino acid (BCAA) concentrations are an independent predictor of type 2 diabetes risk and incidence [86–89], and considering that BCAAs are essential amino acids that cannot be synthesized *de novo*, BCAA concentrations in plasma must reflect either dietary intake and/or an imbalance between skeletal muscle protein anabolism and skeletal muscle protein catabolism. Sarcopenia tips the balance towards the net release of BCAAs into the plasma, and when combined with obesity-associated hyperinsulinemia, could provide a tumor-promoting hormonal and metabolic milieu in the plasma. It should be mentioned that cancer cachexia, in the presence or absence of obesity, likely shares similar metabolic derangements induced by sarcopenia [53,90].

The definition of cachexia differs from sarcopenia based on the underlying cause. Cachexia is wasting of lean mass due to underlying illness, while sarcopenia is lean mass wasting often associated with natural ageing [90]. Cancer-associated cachexia illustrates another concept: cancer *per se* may cause systemic metabolic perturbations in skeletal muscle and other tissues. Tumor-derived inflammatory mediators, including IL-6 [91–93] and TNFα [94–98] have causal roles in tissue-specific insulin resistance, including liver, adipose tissue, and skeletal muscle. In addition, once a tumor grows to a certain size, it is likely that it can compete for nutrients to a similar degree compared with that of other organs: ^18^F-FDG PET/CT data comparing tissue-specific glucose uptake shows that maximal glucose uptake capacity in breast, head and neck squamous cell, soft tissue sarcoma, and non-small cell lung tumors is 3–10 times greater than other organs including skeletal muscle, adipose tissue, and spleen [99]. Thus, in considering the tumor as another fractional contributor to the consumption of circulating metabolites, it is clear that relative sizes of each compartment (vital organs, muscle, adipose tissue, and tumors) can drive relatively large changes in systemic nutrient partitioning.

In sum, there are manifold mechanisms related to excess adiposity, location of adipose tissue, and metabolic derangements independent of body mass that confer risk for metabolic disease, cancer, and all-cause mortality.

## Canonical insulin signaling

**Figure 2.**
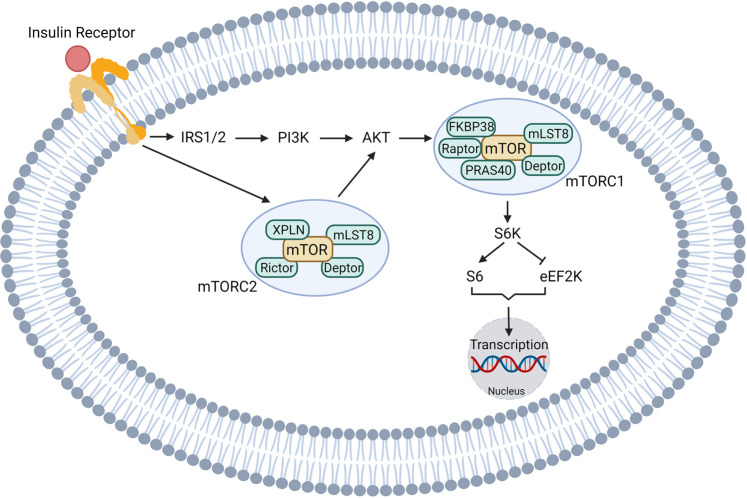
Insulin signaling promotes cell division in tumors. Made in BioRender.com.

Insulin is secreted by beta cells in the pancreatic islet and is recognized by IRs, which are expressed in all cell types in the body, including tumor cells. Canonical insulin signaling pathways in tumor cells are depicted in Figure 2. In support of a critical role for insulin in cancer progression, high IR expression is a poor prognostic factor in lung cancer [100], breast cancer [101], and colon cancer [102]. After ligand binding, IR activates its tyrosine kinase and initiates downstream signaling including the PI3K–AKT [103–105], mTOR [106–108], and RAS–MAPK pathways [105,109,110]. Insulin receptor substrates (IRSs), which are the adaptor proteins of the IR, recruit multiple signaling complexes [111–114]. In particular, growth factor receptor-bound protein 2 (Grb2) is recruited to the binding motif on IRS [115,116], which in turn forms a complex with guanine nucleotide exchange factor Son of Sevenless and phosphorylates RAS. Activated RAS (RAS-GTP) activates the mitogen-activated protein kinase (MAPK) signaling cascade, including extracellular signal-regulated kinase 1/2 (ERK1/2) [117,118]. Rac1, a member of the superfamily of small guanosine triphosphatases [119], is another important signaling pathway downstream of the IR. Rac1 functions as a key regulator of insulin-induced glucose uptake [120,121] and glucose-induced insulin secretion [122]. More importantly, up-regulation of Rac1 is closely related to tumor development in multiple cancer types by promoting cell proliferation and migration as well as angiogenesis [119,123–126]. Taken together, there is no doubt that these kinases promote gene expression in pathways related to cell survival and proliferation [127].

Insulin can also bind to the insulin-like growth factor 1 (IGF1) receptor [116,128,129], which consequently activates the mitogenic signaling pathways that promotes cellular growth and proliferation. Although there is significant redundancy in the intracellular insulin and IGF signaling pathways, some studies imply that they may have distinct roles in malignancies. For instance, Gallagher et al. [130] showed *in vivo* that IR phosphorylation, but not IGF-IR or hybrid receptor phosphorylation, promotes mammary tumor growth in mice with skeletal muscle insulin resistance. These authors also showed that AspB10, an insulin analog that binds specifically to the IR, has a similar effect to increase tumor growth independently of IGF signaling.

However, the IGF signaling pathway also supports the formation and maintenance of cancer stem cells [131–133], which play an important role in the epithelial-to-mesenchymal transition [134,135] and consequent tumor metastasis in both liver cancer [135] and leukemia [136]. Recently, Shahbazi et al. [137] demonstrated that insulin acts as a key stimulator of the mRNA transcriptome, seeding, proliferation, and phosphorylation in human induced pluripotent stem cells (hiPSC). These data highlight a new mechanism by which insulin may promote tumor progression by inducing and enhancing cancer stem cells, leading to tumor growth and metastasis.

## Insulin and tumor cell energetics

Compared with healthy cells, tumor cells have tremendous energy requirements to support proliferation and invasion. Therefore, tumor cells tend to modify their metabolic pattern, exemplified by the transition of the primary glucose utilization pathway from oxidative phosphorylation to glycolysis, i.e. the Warburg effect [138–140]. This metabolic shift not only allows tumor cells to convert nutrients into energy in an oxygen-deprived microenvironment, but also provides building blocks for biosynthesis and cellular proliferation. For instance, glucose-6-phosphate (a glycolytic intermediate) will enter the pentose phosphate pathway to generate ribulose-5-phosphate, a precursor used for DNA and NADPH generation, as well as lipid synthesis [141]. Besides the proliferative and survival effect described previously, insulin also controls whole-body as well as intracellular metabolism by substrate (glucose) partitioning [142]. Aberrant PI3K–mTOR signaling is common in tumor cells. For instance, a hyperactivated mutation in eukaryotic translation initiation factor 4E binding protein 1 (4E-BP1), a downstream effector of mTOR that forms its transcription complex, is commonly observed in the head and neck squamous cell carcinomas [143]. mTOR also alters glucose availability in tumor cells by regulating glucose uptake [144] and glycogenolysis [107]. Glycogen synthase kinase (GSK)-3 can inhibit mTOR signaling via phosphorylation of tuberous sclerosis complex subunit 2 (TSC2). Buller and colleagues showed that impeding activity of the tumor suppressor gene TSC2 resulted in a substantial increase in GLUT1-mediated glucose uptake. This phenotype depended on mTOR activity, suggesting a role for mTOR in modulating cellular glucose metabolism which may translate to tumor cells [145], likely with a varying impact on cell division in different tumor types. Considering that GLUT1 is the primary glucose transporter expressed in most tumor types, including breast [146], lung [147], renal cell [148], colorectal [149], and melanoma [150], and high expression correlates with poorer prognosis of most tumor types found in the Human Protein Atlas (available from www.proteinatlas.org) [151] including breast, cervical, endometrial, ovarian, head and neck, liver, lung, pancreatic, renal, urothelial, and glioma, it is likely that part of mTOR's effect on cancer progression can be attributed to its modulation of glucose uptake and, consequently, metabolism.

However, many of these studies measure only enzymatic activities and nutrient/metabolite concentrations, lacking the gold-standard steady-state isotopic tracer analysis of metabolic fluxes. Without tracers, it is difficult to distinguish between the effects of oncogenic signaling to reprogram tumor metabolism, from the nutrient-dependent, cancer driver-independent, direct effects of metabolic reprogramming on cancer cell division. The use of isotope tracers to assess tumor metabolism will identify metabolic targets of interest within the complex interactions between oncogenic signaling pathways and pathways regulating substrate metabolism. The importance of the interactions between metabolic and oncogenic signaling pathways is highlighted by the fact that, because insulin signaling pathways are not specific to tumor cells, interventions directly targeting IR signaling pathways result in deleterious effects on liver cells, muscle cells, and other tissues. Specifically, treatment with PI3K–AKT–mTOR inhibitors can cause hyperglycemia and, in rare cases, diabetic ketoacidosis due to the effect of these drugs to interfere with systemic insulin signaling [152–160], and hyperinsulinemia resulting from the β-cells’ attempts to normalize blood glucose can limit the efficacy of these agents [104]. However, use of glucose-wasting sodium-glucose cotransporter-2 (SGLT2) inhibitors or low-carbohydrate ketogenic diets can minimize the deleterious effects of PI3K–AKT–mTOR inhibitors on both systemic glucose homeostasis and on tumor growth, at least in rodent models [104]. Several case reports have suggested that the efficacy of SGLT2 inhibitors and low-carbohydrate diets to prevent PI3K inhibitor-induced hyperglycemia may translate to humans [161,162], and a search of the U.S. ClinicalTrials.gov registry on November 21, 2021 revealed three ongoing trials examining the efficacy of adding SGLT2 inhibitors and/or low-carbohydrate diets in patients treated with PI3K inhibitors. Other experimental strategies to indirectly target insulin signaling in combination with other cancer treatments, such as chemotherapy [163] and immunotherapy [32], are also being actively pursued in the clinic, and will be discussed later in this review.

## Epidemiology: hyperinsulinemia and cancer

**Figure 3.**
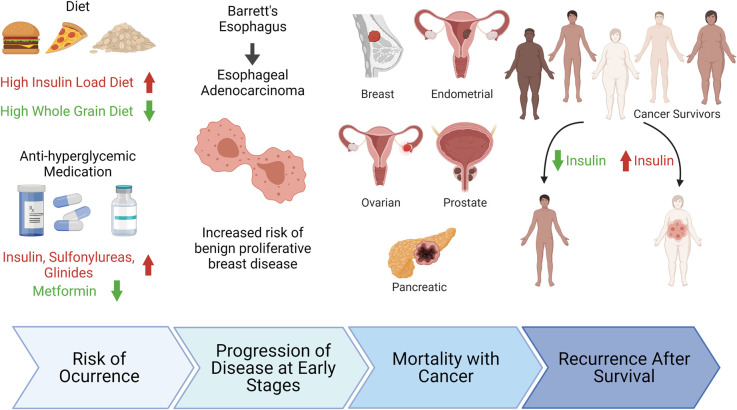
Plasma Insulin is an independent tumor-promoting factor through all stages of cancer progression. Made in BioRender.com.

Hyperinsulinemia is associated with increased risk of breast, endometrial, ovarian [164–166], and prostate cancer [167,168]; increased pancreatic [169] and breast cancer mortality [170]; and increased any cancer mortality [171]. This association holds true in both obese and normal-weight individuals [172], regardless of diabetes, visceral adiposity, or metabolic syndrome status [173]. Hyperinsulinemic dietary patterns are associated with poorer survival and also with increased risk of recurrence in colorectal cancer patients [174–177] and with increased all-cause mortality [178], while high whole-grain and dietary fiber intake lowers the risk of bladder cancer [179] (Figure 3). Other evidence suggests that the influence of insulin on tumor formation has effects in the early stages of cancer, as hyperinsulinemia is independently associated with benign proliferative breast disease [180], and insulin resistance is a risk factor for progressing from Barrett's esophagus to esophageal adenocarcinoma [181]. Evidence for a direct effect of hyperinsulinemia of tumor progression has been suggested by the significantly higher presence of the IR on malignant than benign prostate epithelial cells from human biopsies [182].

In combination with other risk factors including inflammatory markers, sex hormones, and elevated glucose levels, insulin appears to confer independent and perhaps synergistic effects on tumor progression and cancer outcomes.

## Epidemiology: diabetes and cancer

Both type 1 diabetes mellitus (T1DM) and type 2 diabetes mellitus (T2DM) result in hyperglycemia, though their etiologies are strikingly different. T1DM is caused by an autoimmune-mediated destruction of the insulin-producing pancreatic beta cells leading to hyperglycemia through insulinopenia. The lack of endogenous insulin production in T1DM is treated by subcutaneous administration of exogenous insulin. Oftentimes supraphysiologic doses of exogenous insulin are required to suppress endogenous hepatic glucose production and to allow for metabolism of exogenous carbohydrate intake [183]. Endogenous insulin, produced by the pancreas of healthy individuals, can directly enter the portal vein to regulate hepatic glucose metabolism, but this is lacking in those with T1DM. Therefore, to maintain glucose homeostasis as effectively as endogenous insulin does, oftentimes supraphysiologic doses of exogenous insulin are needed. T2DM, on the other hand, is caused by insulin resistance and thus impaired glucose clearance. In an attempt to overcome the inherent insulin resistance, the pancreas increases its insulin production, leading to hyperinsulinemia. The resultant circulating insulin levels in both T1DM, treated with exogenous insulin, and T2DM are substantially higher than those produced by the pancreas of healthy controls. Concomitant hyperinsulinemia in both types of diabetes has now become one of the major proposed mechanisms by which diabetes might promote cancer development, regardless of its origin (i.e. endogenous or exogenous).

In recent years, epidemiologic studies have shown evidence for a higher incidence of various site-specific cancers in people with diabetes mellitus, especially T2DM and to a lesser extent T1DM, compared with the general population. More than a twofold relative risk has been reported for endometrial, hepatic, and pancreatic cancer and an up to 1.5-fold relative risk for bladder, breast and colorectal cancer in T2DM [184–188]. In addition to the higher risk for developing the aforementioned cancers, patients with diabetes reportedly suffer from higher age adjusted short- and long-term mortality rates when diagnosed with cancer [184,189]. The epidemiological association between diabetes mellitus and cancer has led to the investigation of possible mechanistic links between the two as well as between the potential role of diabetes therapeutics in the development of cancer.

One of the major proposed mechanisms by which diabetes might promote cancer development is hyperinsulinemia, regardless of its cause (endogenous or exogenous). The link between insulin and cancer is the topic of this review, but we recognize the high likelihood that insulin is not the only link between obesity, diabetes, and cancer. Additional proposed cancer-promoting factors, especially in the conjunction with concomitant obesity, are hyperglycemia, hyperlipidemia as well as increasing circulating levels of leptin, estrogen, resistin, and inflammatory cytokines along with reduced concentrations of IGF binding proteins and adiponectin levels [190] which are proposed to play a permissive role in tumor cell proliferation, dissemination, and oncogene expression [191].

In addition to diabetes, anti-diabetes therapy has also been implicated in the development of cancer [5]. The list of agents includes incretin analogs, such as GLP-1 receptor agonists, incretin enhancers, such as dipeptidyl peptidase-4 inhibitors, insulins like glargine, along with pioglitazone, and sulfonylureas. All of which have been associated with cancer pathogenesis due to their enhancement of circulating insulin levels. However, many of the studies performed were flawed by inadequate methodology, *in vitro*/*in vivo* conditions that were not concordant/congruent with actual physiology and/or significant bias in study design and data interpretation, such as prevalent-user bias, immortal time bias, and time-lag bias/confounding by indication. Furthermore, many studies did not account and adjust for many covariates, such as disease duration, and severity, amongst others.

Despite the highly suggestive association between diabetes and cancer [184–188,192–201], the underlying molecular and mechanistic links still remain fairly obscure. In addition, it is possible that there is no linear, direct causality between diabetes and cancer, but the link might rather be as multifactorial as the pathology of diabetes itself. For instance, mutuality between diabetes and cancer might be attributable to their common predisposing factors, such as unhealthy lifestyles, including physical inactivity and excess caloric intake, higher adipose mass and decreased lean muscle mass as well as ageing itself. Therefore, further research is needed to identify exact underlying mechanistic causality and identify novel therapeutic and interventional targets.

## Hyperinsulinemia as a therapeutic target in animals with cancer

**Figure 4.**
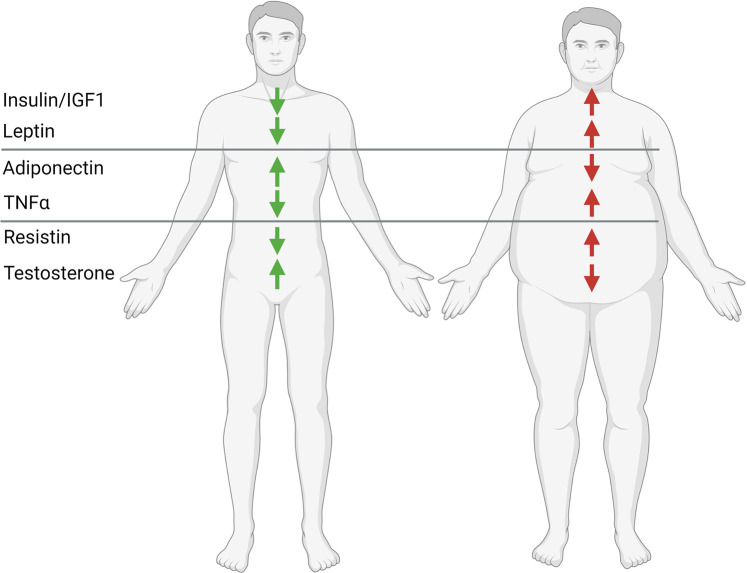
Proposed mechanisms by which obesity may promote the progression of certain tumors. Made in BioRender.com.

Despite mechanistic uncertainties, considering the strong epidemiologic evidence in support of a pathogenic link between insulin and cancer, numerous *in vivo* preclinical studies have explored this possibility interventionally. Both endogenous hyperinsulinemia [202] and exogenous insulin injection [203] promote colorectal cancer growth in rats. Similarly, insulin injection also promotes the progression of pancreatic cancer in Syrian hamsters [204], as well as the development and metastasis of breast [205–207] and colon cancer in mice [208]. To be noted, insulin did not significantly alter body composition in these studies, consistent with a direct impact of insulin to accelerate tumor growth. Preclinical *in vivo* rodent studies have demonstrated that hyperinsulinemia can activate not only its cognate IR but can also bind to and hence stimulate the insulin-like growth factor 1 receptor (IGF1R). Additionally, hyperinsulinemia also promoted increased production of IGF-1 by the liver which in turn further amplified IGF1R signaling through the PI3K–AKT–mTOR and RAS–MAPK pathways, which stimulate expression of the MYC proto-oncogene, cell proliferation, anti-apoptotic, and anabolic effects in tumor cells [104,190,209–212]. Of course, hyperinsulinemia is not the only mechanistic link between obesity and cancer. Although discussion of these alternative mechanisms is beyond the scope of this review, we acknowledge that insulin-independent mechanisms — for example, lipid peroxidation and metabolism [213], fibroblast growth factor receptor-1 [214], creatine [215], leptin [216], inflammatory cytokines [217], and many others which space limitations do not permit us to discuss in any detail (Figure 4) — play a key role in the progression of obesity-associated cancers as well.

## Hyperinsulinemia as a therapeutic target in patients with cancer

Several epidemiologic studies have correlated antihyperglycemic medication use with risk or outcomes of cancer, and have generally concluded that patients with type 2 diabetes treated with insulin and with sulfonylureas, which stimulate insulin secretion, have higher cancer incidence and mortality than those treated with metformin [218–223]. However, these results do not uniformly point to a direct link between insulin and cancer: despite strong preclinical evidence [208,224,225], the addition of metformin to chemotherapy for non-small cell lung cancer reduced hyperinsulinemia, but did not provide survival benefit [226]. In this same study, however, patients with high ^18^F-Fluorodeoxyglucose uptake on their PET scans received a mortality benefit from metformin, suggesting certain glucose-dependent but insulin-independent effects in the tumor microenvironment. In addition, metformin reduced the risk of cancer in a type 2 diabetic population, with no differences in fasting insulin or the homeostatic model assessment for insulin resistance (HOMA-IR), but the metformin group had less exogenous insulin use [227]. Another study found that use of insulin, glinides, and sulfonylureas increased the risk for gastrointestinal and lung cancers [220]. Clearly, more evidence is required in humans to determine whether and how the reversal of hyperinsulinemia can slow tumor growth and improve clinical outcomes.

Likely, it is of particular importance to develop and apply strategies to enhance systemic metabolism during cancer treatment because of the effects of standard-of-care therapies to induce metabolic dysfunction. In addition to the effect of immune checkpoint inhibitors to cause autoimmune diabetes, which will be discussed later in this review, chemotherapy commonly causes weight gain and insulin resistance [228]. Moreover, insulin resistance is a predictor of poor outcomes in those treated with chemotherapy: a recent study found the probability of a pathological complete response to treatment for breast cancer to be close to five times lower in those with insulin resistance, as compared with those without [229], with other studies generating similar conclusions regarding the detrimental effect of chemotherapy on metabolic health in multiple tumor types [230–234]. Although less commonly studied, androgen deprivation therapy [235], radiotherapy [236–238], and stem cell transplant [239], as well as chemotherapy in combination with radiotherapy [240], also appear to pose an increased risk of excess weight and impaired insulin sensitivity in cancer survivors. However, surprisingly little work has focused on the mechanisms by which these standard-of-care cancer treatments may lead to metabolic dysfunction. Historically, chemotherapy was commonly administered in 5% glucose water; although normal saline is increasingly preferred, the epidemiologic data may recommend a more robust examination of this common practice, considering the extra carbohydrate load presented by glucose diluent and its potential impact on systemic metabolism during chemotherapy treatment.

## Exercise and cancer

Exercise is a well-established insulin-sensitizing intervention. Studies dating back at least 100 years [241] have demonstrated that both acutely and chronically, aerobic exercise has the capacity to reduce plasma glycemia and enhance insulin action in skeletal muscle, in both an intensity- and duration-dependent manner [242–262]. Exercise research has been fundamental in understanding glucose transport, and exercise was used as a model to illustrate insulin-independent (GLUT4-dependent) skeletal muscle glucose uptake, making exercise prescription in patients with insulin resistance an appealing therapeutic modality [263,264]. As the links between insulin resistance and cancer have emerged over the past several decades, exercise quickly became a standard adjuvant for cancer therapy [265–273].

Basic and translational studies have consistently shown immense effects of exercise (most commonly voluntary wheel running in rodents) to slow tumor growth [274–282] and reduce metastases [283–286] in tumor-bearing animals. Multiple mechanisms have been suggested to mediate exercise's anti-cancer effects: enhanced angiogenesis and thus increased immune cell infiltration [280,284], forced-swimming-induced catecholamine induction has been suggested to enhance natural killer cell infiltration into tumors [282,287], and exercise training induced improvements in insulin resistance [288,289], and thus reductions in tumor anabolism, among others, have been suggested.

As would be predicted by the epidemiologic and clinical data, exercise exerts a modest but significant effect to reduce cancer risk and slow tumor progression [290–294]; however, whether and to what extent the beneficial effect of exercise is mediated by reversal of hyperinsulinemia *per se* and to what extent this effect is reliant on alterations in tumor and/or immunometabolism is an open question. The PreHAB study, where obese women with breast cancer were randomized to a combination of aerobic and resistance exercise training program, compared with a mindfulness control group, for 4 weeks prior to surgical excision, demonstrated that exercise reduced circulating insulin, IGF1, and leptin, though only leptin reductions were significantly different from control patients [295]. Future clinical trials on the impact of exercise on tumor biology should continue to collect biomarker data to provide further insight into the mechanistic basis of exercise and cancer interactions. In addition, the frequency, intensity, type, and duration of exercise necessary to induce beneficial metabolic and anti-tumor effects in patients is unknown and largely unexplored.

A topic of recent interest involves how exercise may alter immune function through metabolic reprogramming. Work primarily derived from RNA sequencing and metabolomics has suggested that different substrates play key regulatory roles in immune action. Conventional wisdom holds that glucose and glutamine metabolism may be crucial to promote differentiation, activation, and clonal expansion [296–306], while fatty acid metabolism may also play a key role in immune cell longevity, including preventing exhaustion in T cells and dendritic cells [307,308] and promoting regulatory and memory T cell formation and survival [303,307,309–314]. These data would predict that approaches to transiently increase systemic glucose metabolism in a cyclic pattern, while chronically increasing fatty acid metabolism, would be most beneficial in improving anti-cancer immunity.

Aerobic exercise is a classic means of inducing just these changes. Acutely upon initiation of intense exercise, there is a shift to glucose metabolism [315–317]; however, during recovery from exercise and during exercise training, a shift in whole-body metabolism to increase fatty acid oxidation has been repeatedly observed [318–320]. As discussed in the next section, it is possible — though not yet proven — that both alterations may yield anti-cancer benefits by their actions on immune cells.

## Cancer immunology, nutrients, and insulin

With rising recognition of the interactions between insulin, substrates, and tumor progression, dietary modifications represent an area of burgeoning interest in cancer therapeutics. The impact of diet on tumor metabolism and prognosis is likely nuanced: while a low-carbohydrate, ketogenic diet reduces tumor glucose uptake in human patients [321–323], a high fat, low-carbohydrate diet — seemingly paradoxically — increases tumor glucose uptake in rodents [207,208,324]. In addition to potential species differences, studies in this vein are plagued by a critical confounder: there is wide variance in both adherence to any diet, and in the total caloric load ingested on most diets, leading to discrepancies in dietary intervention studies in terms of whether participants experience a positive, negative, or neutral energy balance. These differences in diet-induced alterations in energy balance across various studies may lead to differences in the effect of the diet on insulin responsiveness.

Additionally, recent research has highlighted the possibility that various diets may affect cancer outcomes in a tumor cell-autonomous manner: by affecting the immune response to cancer. Insulin has been implicated in the modulation of different immune phenotypes and responses [325], as evidenced by the expression of insulin receptors (IRs) on T, B cells and macrophages after activation [326,327]. Furthermore, conditional knockdown of these IRs has been found to reduce aerobic glycolysis, which is evidenced in the decreased expression of GLUT 3,4 and the reduction in lactate production [328], all of which are hallmarks of the Warburg effect of cancer. The immunomodulatory role of insulin has been mechanistically linked to the PI3K/Akt/mTOR signaling pathway; binding of insulin to an IR results in its dimerization and autophosphorylation, which results in the activation of IRSs [329,330]. These activated IRSs in-turn stimulates PI3K resulting in the phosphorylation of AKT at tyrosine-308 by PDK1 and at Serine-473 by mTORC2. Interestingly, PI3K/Akt/mTOR signaling pathway is also often dysregulated in many cancer pathologies. Furthermore, IR-deficient T cells have been found to have decreased expression of Myc, which is a transcription factor that is downstream of the PI3K/Akt/mTOR signaling pathway and it is involved in glycolytic metabolism [329,331]. Myc is also an oncogene, whose dysregulation results in the propagation of many cancer pathologies [332].

Whether insulin-dependent or -independent, it is clear that substrate metabolism also plays a role in immune function and longevity. It has been suggested that metabolic competition in the TME is a key mechanism by which immune cells limit tumor growth [296,297,333]. While this possibility could be dismissed offhand by considering the much greater biomass of tumor cells versus immune cells in a typical tumor, it is important to consider the primary glucose transporters expressed by each cell type. While as mentioned earlier GLUT1 is the primary glucose transporter expressed in most tumor types, the higher-affinity GLUT3 is the primary glucose transporter expressed by T cells, and data from the open-access Immunological Proteome Resource (ImmPRes) show that its expression is markedly increased in activated T cells. As the *K*_m_ of GLUT1 is 7–26 mM [334,335] while the *K*_m_ of GLUT3 is less than 2 mM [335], GLUT3 is better able to facilitate glucose uptake at the low glucose concentrations characteristic of the TME, suggesting that tumor-T cell competition for glucose may be relevant in determining cancer prognosis.

In addition, exercise is another well-studied modulator of systemic and tissue-specific nutrient partitioning. A single bout of exercise stimulates whole-body glucose metabolism by increasing both insulin-mediated and insulin-independent glucose uptake in tissues [245,247,249,253,336–343]. During acute exercise, the exercising muscle and, therefore, whole organism rely first on breakdown of glycogen, the short-term storage form of glucose. Once endurance exercise is sustained for longer durations (beyond ∼10 min), *de novo* glucose synthesis (gluconeogenesis) increases to enable systemic increases in rates of aerobic and non-aerobic glucose metabolism [344,345]. This rapid increase in systemic reliance upon glucose is made possible by a 2–3-fold induction of hepatic, and possibly renal, glucose production [249,340–342]. As glucose is considered the primary driver of T cell activation [306,346–350], exercise-induced increases in glucose metabolism could mediate the effect of exercise to promote cytotoxic T cell function in mice with cancer.

However, chronically, exercise rehabilitation and training reduce the whole-organism respiratory exchange ratio, reflecting a shift in systemic metabolism from oxidation of glucose to oxidation of fatty acids [351–356]. This increased reliance on fatty acids has important implications for all-cause mortality: a lower respiratory exchange ratio is associated with a lower incidence of postoperative complications [357–359] and improved survival in patients with sepsis [360,361] and heart failure [362–364] as well as in ageing mice [365], highlighting the intriguing possibility that chronically increased systemic fatty acid metabolism may improve outcomes in other conditions in which the immune system is important, including cancer.

While glucose metabolism has received most of the attention paid to cancer immunometabolism, increasing evidence suggests that fatty acid metabolism should not be ignored. Metabolic flexibility appears to be critical in promoting cytotoxic effector function while also preserving long-term immune cell health and longevity, with glucose and glutamine metabolism promoting effector function [296,297,366–370] and fatty acid metabolism predominantly fueling naïve T cell metabolism [298,371–373], T_reg_ formation [313], memory T cell formation and survival [307,312,314,374–377], and natural killer cell [378] and dendritic cell maturation and function [379]. Systemic metabolic inflexibility — that is, a constant and exclusive reliance upon either glucose or fatty acid metabolism would, then, be predicted to worsen outcomes: excessive reliance on glucose may acutely promote effector function but chronically promote exhaustion and worsen memory cell formation, whereas excessive reliance on fatty acids may enhance longevity but worsen effector function.

In considering possible targets to mimic the effect of exercise on anti-cancer immune function, carnitine palmitoyltransferase I (CPT1) represents an attractive target. CPT1 is considered the gatekeeper for mitochondrial fatty acid oxidation, as it catalyzes the formation of acylcarnitines for transport from the cytosol into the mitochondria. Chronic exercise increases CPT1 expression in skeletal muscles and peripheral blood mononuclear cells of rodents [380–386] and humans [387–390]; however, future studies will be required to determine the functional relevance of this increase in CPT1 expression on anti-cancer immune function *per se*.

Given these links between insulin, immune function and cancer, including the exhaustive evidence provided for the connections between obesity, inflammation, insulin-dependent diabetes, and cancer, this serves the logical thinking that there are links between cancer immunology and insulin. Though this field is relatively understudied, there are certain lines of evidence that could invigorate future research work. One line of such evidence described in 2015, follows the use of anti-programmed cell death-1 (PD-1) therapies such as pembrolizumab in a patient with BRAF wild-type cutaneous melanoma that subsequently developed autoimmune diabetes [391]. Pembrolizumab is an immune checkpoint inhibitor and an IgG4 monoclonal antibody that targets PD-1 [391]. Interestingly, immune checkpoint inhibitors such as pembrolizumab seem to modulate the same nodal networks that are involved insulin signaling [331]. Though the ability of immune checkpoint inhibitors to induce autoimmune diabetes has been well described, this serious adverse effect of immune checkpoint inhibitors is extremely rare at ∼1% of those treated with ICIs for cancer [392].

Though a clear mechanistic relationship between immune checkpoint inhibitors and the subsequent presentation of autoimmune diabetes is yet to be described, some inferences can be made. CD28 is a co-activator for T cell function and similar to the IR activation of the PI3K/Akt/mTOR signaling pathway described above, tyrosine phosphorylation of the cytoplasmic tail of CD28 up-regulates the activity PI3K/Akt signaling in T cells [393]. Given that activation of PD-1 directly and CTLA-4 indirectly antagonizes the up-regulation of PI3K/Akt signaling, it is quite possible that efficacy of immune checkpoint inhibitors is tied to insulin signaling.

## Concluding thoughts

Although many studies, including from our group, have attempted to draw linear relationships between obesity, diabetes, hyperinsulinemia, and cancer, and have assessed the links between these devastating conditions in isolation, it is likely that the relationships between these conditions are more complex than that. While scientific rigor requires one to choose a target of interest and probe it as independently as possible, *in vivo* there is undoubtedly interplay between insulin and many other tumor-promoting or -limiting factors. However, this does not undercut the potential for insulin-targeting therapies to serve as a useful adjunct to standard-of-care therapies in cancer. What is unarguably clear at this time is that there is a link between altered systemic metabolism and cancer. Future studies to mechanistically understand this link are of critical importance as we stand on a precipice of continuing increases in rates of both obesity, diabetes, and cancer in the U.S. and worldwide.

## Abbreviations

BCAA: branched-chain amino acid
CPT1: carnitine palmitoyltransferase I
CT: computed tomography
IGF1: insulin-like growth factor 1
IGF1R: insulin-like growth factor 1 receptor
ImmPRes: immunological proteome resource
IR: insulin receptor
IRSs: insulin receptor substrates
MAPK: mitogen-activated protein kinase
SGLT2: sodium-glucose cotransporter-2
T1DM: type 1 diabetes mellitus
T2DM: type 2 diabetes mellitus
